# Supplementary material for: Late Paleozoic oxygenation of marine environments supported by dolomite U-Pb dating
Source: Nat Commun. 2024 Apr 3;15:2892. doi: 10.1038/s41467-024-46660-7 (PMC10991507; doi:10.1038/s41467-024-46660-7)
Supplement: Supplementary file 1 — Supplementary Information [file 41467_2024_46660_MOESM1_ESM.pdf]

Late Paleozoic Oxygenation of Marine Environments Supported by Dolomite U-Pb Dating

Supplementary Information

Note 1 - Analytical solution for the calculation of average initial $^{238}U/^{206}Pb$ in dolomite precursory minerals.

Figure S1 - Tera-Wasserburg plots of new and reprocessed data.

Note S1 – Analytical solution for the calculation of average initial $^{238}U/^{206}Pb$ in dolomite precursory mineral

Observed initial $^{206}Pb/^{204}Pb$ in the dolomite sample is considered to evolve from the terrestrial value at the time of deposition (defined by the Stacey and Kremers [1] two-stage model) during the interval between deposition and alteration (as represented by the U-Pb date and depositional age of the dolomite and its host strata, respectively):

$$\left[\frac{206}{204}\right]_{dol,PD} = \left[\frac{206}{204}\right]_{pre,0} + \left[\frac{238}{204}\right]_{pre,0} (1 - e^{-\lambda_{238}\Delta t}) \qquad \text{eq. 1}$$

where 'dol,PD' is for initial values measured in dolomites at the present day; 'pre,0' marks values in the dolomite-precursory phase at the time of deposition. 'pre,0' lead isotope $^{206}Pb/^{204}Pb$ or $^{207}Pb/^{204}Pb$ ratios are based on Stacey and Kremers (1975) values at the depositional age. Δt is the difference between depositional age and U-Pb date.

In a similar way we write:

$$\left[\frac{207}{204}\right]_{dol,PD} = \left[\frac{207}{204}\right]_{pre,0} + \left[\frac{235}{204}\right]_{pre,0} (1 - e^{-\lambda_{235}\Delta t}) \qquad \text{eq. 2}$$

The ratio $\left[\frac{235}{204}\right]_{pre,0}$ is calculated at the depositional age (t_0):

$$\left[\frac{235}{204}\right]_{pre,0} = \left[\frac{238}{204}\right]_{pre,0} \frac{137.818}{e^{(\lambda_{238}-\lambda_{235})t_0}} \qquad \text{eq. 3}$$

Dividing eq. 2 by eq. 1 we get $\left[\frac{207}{206}\right]_{dol,PD}$ in the dolomite:

$$\left[\frac{207}{206}\right]_{dol,PD} = \frac{\left[\frac{207}{204}\right]_{pre,0} + \left[\frac{235}{204}\right]_{pre,0} (1 - e^{-\lambda_{235}\Delta t})}{\left[\frac{206}{204}\right]_{pre,0} + \left[\frac{238}{204}\right]_{pre,0} (1 - e^{-\lambda_{238}\Delta t})} \qquad \text{eq. 4}$$

Substituting eq. 3 for $\left[\frac{235}{204}\right]_{pre,0}$ in eq. 4 and rearranging we obtain:

$$\left[\frac{238}{204}\right]_{pre,0} = \frac{\left[\frac{207}{206}\right]_{dol,PD} \left[\frac{206}{204}\right]_{pre,0} - \left[\frac{207}{204}\right]_{pre,0}}{\frac{137.818(1 - e^{-\lambda_{235}\Delta t})}{e^{(\lambda_{238}-\lambda_{235})t_0}} - \left[\frac{207}{206}\right]_{dol,PD} (1 - e^{-\lambda_{238}\Delta t})} \qquad \text{eq. 5}$$

And finally the 238/206 in the dolomite precursor mineral equals

$$\left[\frac{238}{206}\right]_{pre,0} = \frac{\left[\frac{238}{204}\right]_{pre,0}}{\left[\frac{206}{204}\right]_{pre,0}} \qquad \text{eq. 6}$$

Supplementary References

1. Stacey, J. S. & Kramers, J. D. Approximation of terrestrial lead isotope evolution by a two-stage model. *Earth and Planetary Science Letters* **26**, 207–221 (1975).

Figure S1 – Sixty-five Tera-Wasserburg plots for new and reprocessed U-Pb isotope data. Panel labels correspond to sample names in Table S1. Blue curve represents the Concordia. Semi-transparent ellipses are 2SD covariance error of single points measured in a dolomite fabric. Black lines are best-fit linear trend. Gray lines represent the 2SD uncertainty of the best-fit trend based on a York analysis, which propagates to uncertainty in U-Pb date and initial $^{207}Pb/^{206}Pb$. Red dots are the intercepts between the best fit and 2SD uncertainty trends and the Concordia.

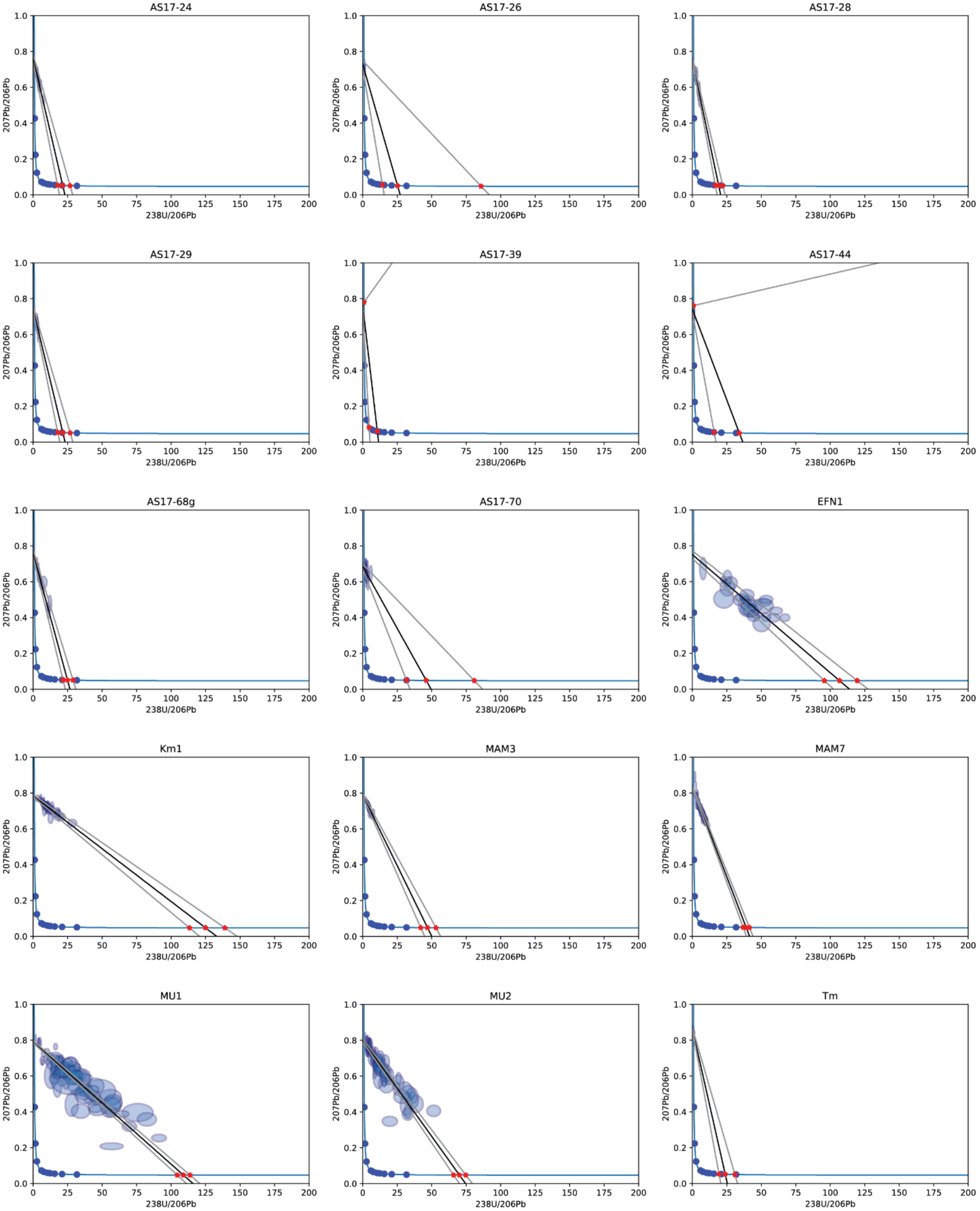

AS17-24
AS17-26
AS17-28
AS17-29
AS17-39
AS17-44
AS17-68g
AS17-70
EFN1
Km1
MAM3
MAM7
MU1
MU2
Tm
207Pb/206Pb
238U/206Pb

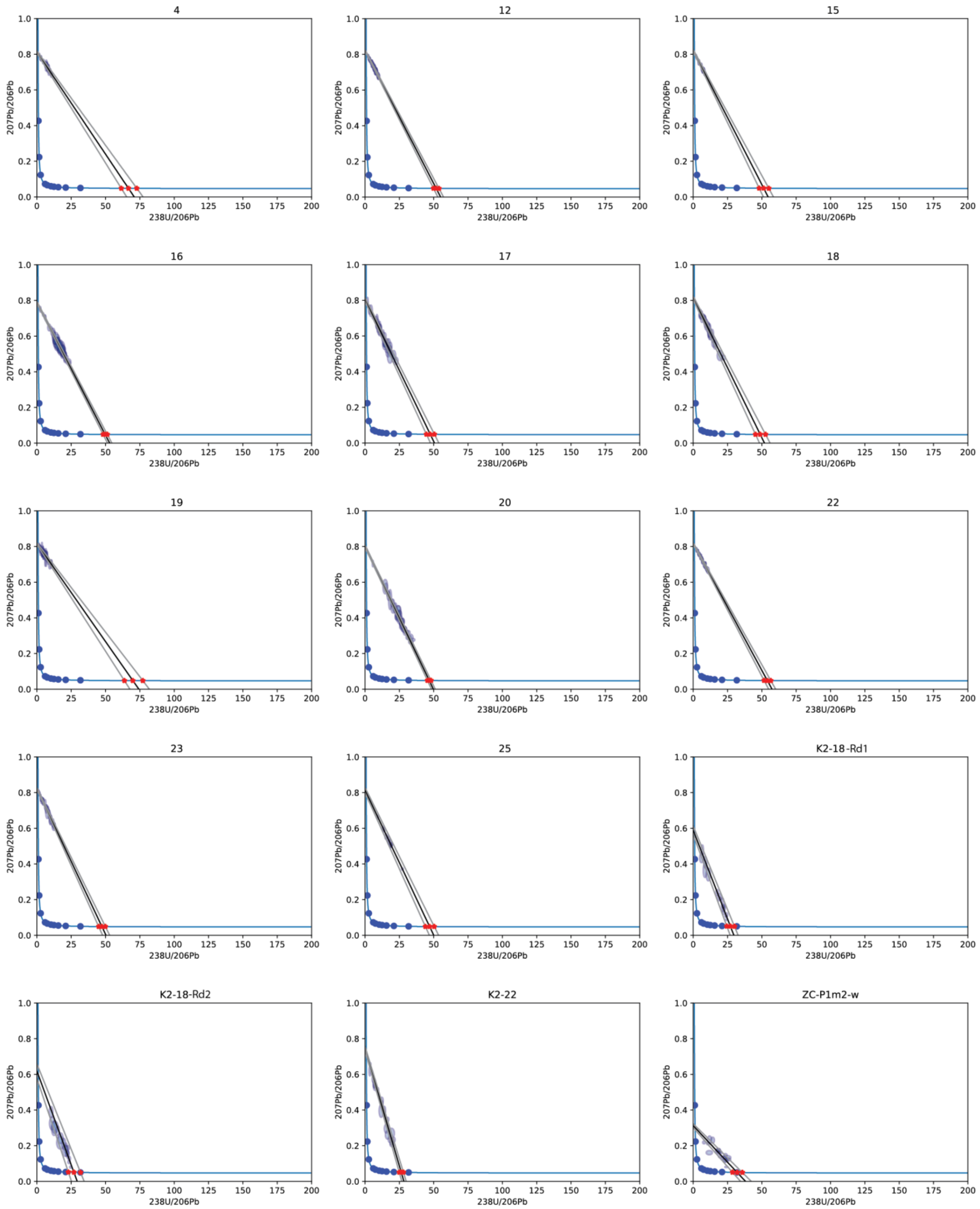
4
12
15
16
17
18
19
20
22
23
25
K2-18-Rd1
K2-18-Rd2
K2-22
ZC-P1m2-w
207Pb/206Pb
238U/206Pb

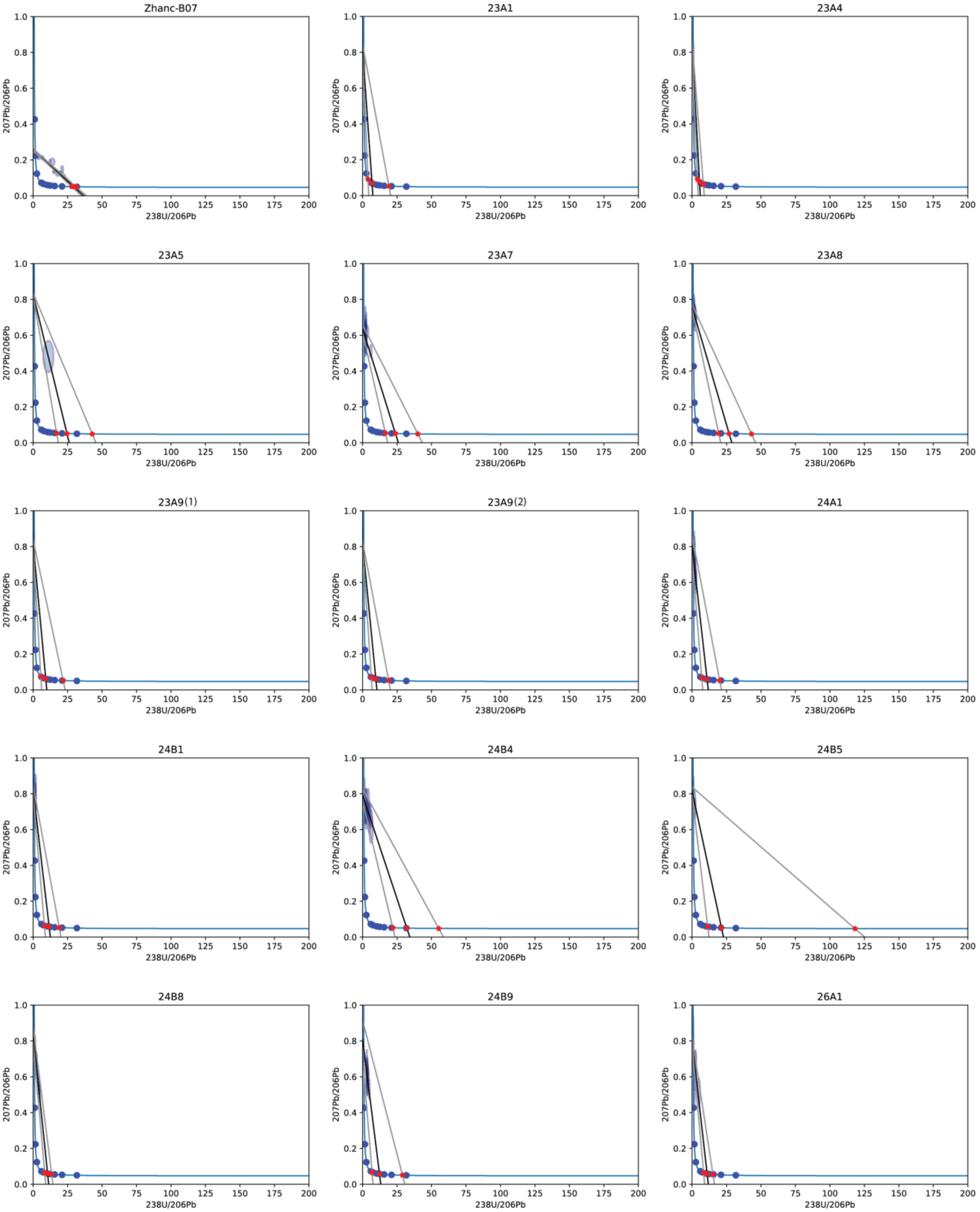
Zhanc-B07
23A1
23A4
23A5
23A7
23A8
23A9(1)
23A9(2)
24A1
24B1
24B4
24B5
24B8
24B9
26A1
207Pb/206Pb
238U/206Pb

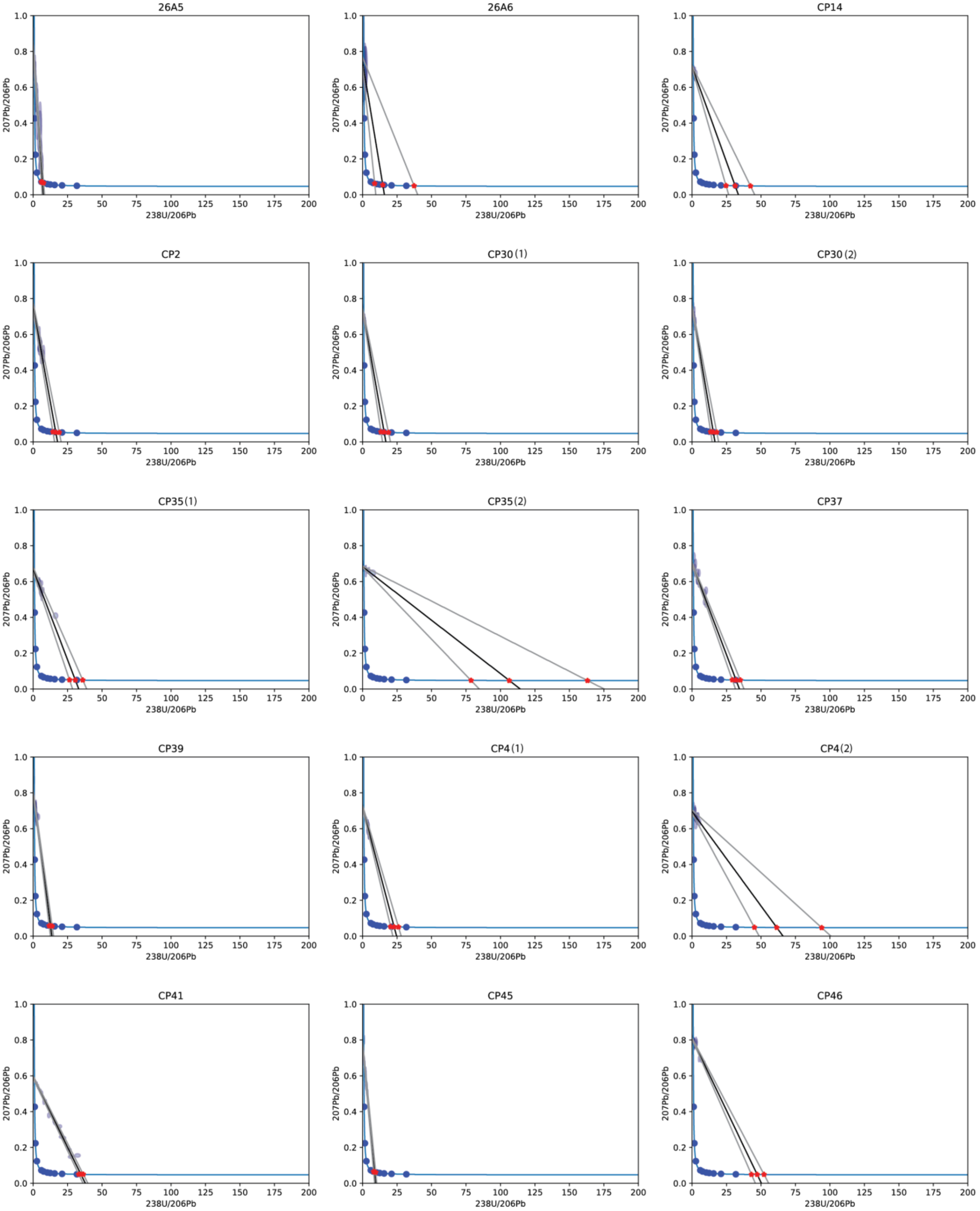
26A5
26A6
CP14
CP2
CP30 (1)
CP30 (2)
CP35 (1)
CP35 (2)
CP37
CP39
CP4 (1)
CP4 (2)
CP41
CP45
CP46
207Pb/206Pb
238U/206Pb

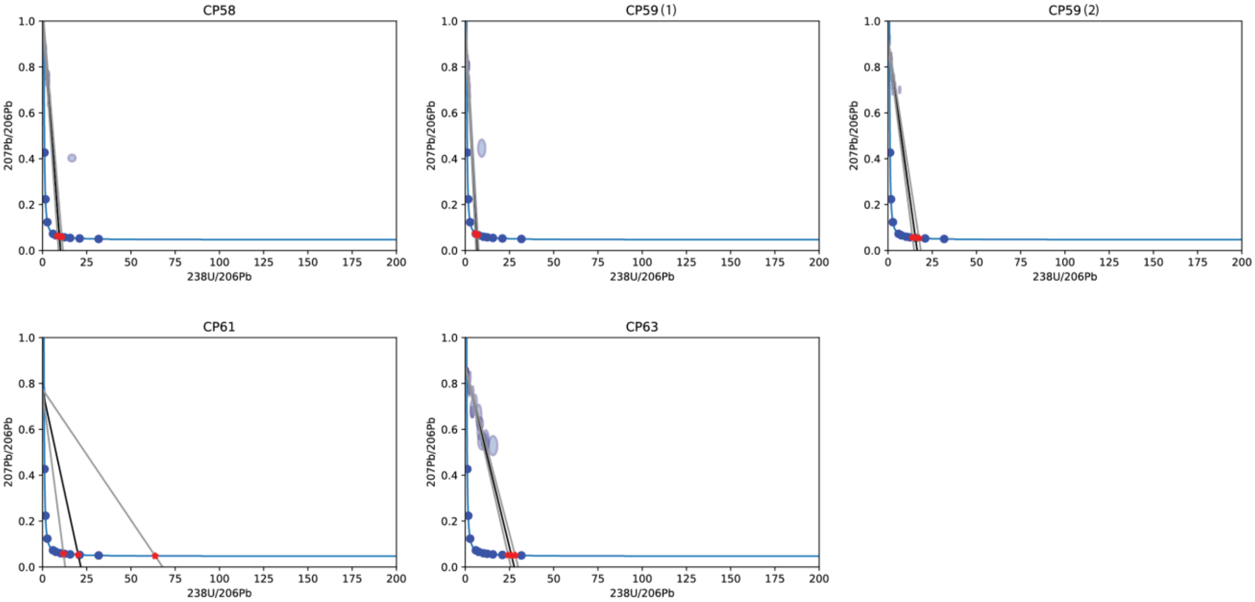
CP58
CP59 (1)
CP59 (2)
CP61
CP63
207Pb/206Pb
238U/206Pb
1.0
0.8
0.6
0.4
0.2
0.0
0
25
50
75
100
125
150
175
200
