# Supplementary material for: Late Paleozoic oxygenation of marine environments supported by dolomite U-Pb dating
Source: Nat Commun. 2024 Apr 3;15:2892. doi: 10.1038/s41467-024-46660-7 (PMC10991507; doi:10.1038/s41467-024-46660-7)
Supplement: Supplementary file 2 — Description of Additional Supplementary Information [file 41467_2024_46660_MOESM2_ESM.pdf]

Description of Additional Supplementary Files

File name: Supplementary Data 1

Description: Summary of sample descriptions, stratigraphic ages, U-Pb dating data, and calculated initial 238U/206Pb for new and compiled dolomite samples.

File name: Supplementary Data 2

Description: Summary of sample location (coordinate and stratigraphic units) analyzed in this study.

File name: Supplementary Data 3

Description: Raw U-Pb data measured in this study.
